# Supplementary material for: What Is the Significance of Indeterminate Pulmonary Nodules in High-Grade Soft Tissue Sarcomas? A Retrospective Cohort Study
Source: Cancers (Basel). 2023 Jul 7;15(13):3531. doi: 10.3390/cancers15133531 (PMC10340540; doi:10.3390/cancers15133531)
Supplement: Supplementary file 1 [file cancers-15-03531-s001.zip › cancers-2371552-supplementary.pdf]

Supplementary Table S1: Clinical details of patients with IPNs at diagnosis progressing to lung metastases. * indicates still alive.

Age at Diagnosis	Site of Primary	Histological Subtype	Grade	Max Tumour Dimension (cm)	Depth Relative to the Fascia	More than 1 IPN?	Bilateral Distribution?	IPN 5 mm or Greater in Size?	Time to Progression (days)	How was Progression of IPN Detected	Lung Metastasis Resected?	Survival (days)
68	Lower limb	Synovial sarcoma	2	32	Deep	Yes	Yes	Yes	13	Progression detected on CTPA for post-operative PE	No	231
84	Lower limb	leiomyosarcoma	3	10	Deep	Yes	Yes	Yes	146	Progression identified on interval CT at 3 months	No	381
83	Lower limb	UPS	3	12	Deep	Yes	No	Yes	479	Identified on CXR when admitted unwell, confirmed with CT	No	570
62	Lower limb	leiomyosarcoma	3	8	Deep	Yes	Yes	No	444	Identified on routine surveillance CXR, confirmed with CT	No	783
87	Upper limb	Rhabdomyosarcoma	3	5	Superficial	Yes	Yes	No	132	Progression identified on interval CT at 3 months	No	252
66	Lower limb	Rhabdomyosarcoma	3	10	Deep	Yes	Yes	Yes	54	Progression seen on CTPA conducted during post operative stay for shortness of breath	No	67
76	Lower limb	UPS	3	19	Superficial	No	-	No	285	Identified on routine surveillance CXR, confirmed with CT	No	710
67	Lower limb	Liposarcoma	3	14	Deep	No	-	Yes	80	Progression identified on interval CT at 3 months	No	244
84	Lower limb	UPS	3	9.7	Deep	Yes	No	Yes	143	Progression identified on interval CT at 6 months	No	203
59	Lower limb	Leiomyosarcoma	3	9.5	Deep	Yes	Yes	No	238	Progression identified on restaging CT for liver metastases	No	602
63	Lower limb	Leiomyosarcoma	3	10	Deep	No	-	No	383	Progression identified on interval CT at 12 months	Yes	798
76	Lower limb	UPS	3	6.5	Superficial	No	-	No	299	Progression identified on restaging CT for groin metastases	No	327
11	Lower limb	Alveolar Soft Parts	2	2.7	Deep	Yes	Yes	No	103	Progression identified on interval CT at 3 months	Yes	2252 *
63	Lower limb	MPNST	3	20	Deep	No	-	Yes	209	Progression identified on interval CT to monitor response of other mets to chemo	No	209
32	Lower limb	UPS	3	11.4	Deep	Yes	Yes	Yes	83	Progression identified on interval CT at 3 months	No	83
69	Lower limb	Leiomyosarcoma	3	9.3	Deep	Yes	No	No	131	Identified on routine surveillance CXR, confirmed with CT	No	445
52	Chest wall	MPNST	3	7.3	Deep	Yes	Yes	Yes	146	Identified on CT when admitted for breathlessness	No	189
73	Lower linb	UPS	3	12	Deep	No	-	Yes	171	Identified on routine surveillance CXR, confirmed with CT	No	185
55	Lower limb	Rhabdomyosarcoma	3	14.5	Deep	Yes	Yes	Yes	66	Identified on CT when admitted for breathlessness	No	66
75	Breast	Angiosarcoma	3	24.5	Superficial	Yes	No	No	18	Identified on CT for new pleural effusion	No	23
56	Lower limb	UPS	3	13.3	Deep	Yes	Yes	Yes	73	Identified on routine surveillance CXR, confirmed with CT	No	112
69	Breast	Angiosarcoma	3	1.1	Superficial	No	-	Yes	489	Progression seen on CTPA for potential PE	No	554
57	Lower limb	UPS	3	4.3	Superficial	No	-	No	260	Progression identified on restaging CT for recurrence	No	511
69	Chest wall	UPS	3	12.5	Superficial	No	-	Yes	22	Progression seen on CT for persistent post-op pleural effusion	No	34
70	Chest wall	UPS	3	14	Deep	Yes	Yes	Yes	88	Progression seen on interval CT at 2 months	178	
67	Trunk	UPS	3	16.5	Deep	Yes	Yes	Yes	557	Identified on routine surveillance CXR, confirmed with CT	No	840
80	Lower limb	UPS	3	5.8	Deep	Yes	Yes	No	126	Progression identified on CT following pleural effusion on CXR	No	182
